# Supplementary material for: Are carbon emissions trading and green financial instruments synergistic? -Comprehensive quantitative research based on content analysis
Source: PLoS One. 2024 Mar 7;19(3):e0298601. doi: 10.1371/journal.pone.0298601 (PMC10919601; doi:10.1371/journal.pone.0298601)
Supplement: S1 Data — (ZIP) [file pone.0298601.s001.zip › Data/地级市Z_F工作报告.zip!/║╙─╧/╫ñ┬φ╡Ω╩╨/└┤╫╘╣1⁄2╓┌║┼í╛┬φ┐╦╩2╛▌═°í┐_2017.docx]

一、过去五年工作回顾侯卫东官场笔记

本届政府任期的五年，是正阳发展史上极具重要意义的五年。在市委、市政府和县委的正确领导以及县人大、县政协的监督支持下，县政府紧紧抓住发展这个第一要务，凝心聚力，务实重干，取得了显著成绩，全县经济呈现出了持续发展的良好态势。2016年，全县生产总值达到159.43亿元，与2011年相比，年均增长8.4%；规模以上工业增加值完成39.18亿元，年均增长13.0%；固定资产投资完成134亿元，年均增长19.8%；社会消费品零售总额完成66.4亿元，年均增长14.3%；财政一般预算收入完成5.41亿元，年均增长19.6%；财政一般公共预算支出40.49亿元，年均增长18.2%；金融机构各项存款余额210.5亿元，年均增长18%；各项贷款余额82.89亿元，年均增长21.5%；城镇居民人均可支配收入和农民人均纯收入分别达到20396元和9709元，年均分别增长8.6%和11%。经济结构进一步优化，产业结构由2011年的40:32.4:27.6调整优化到2016年的32.1:27.5:40.4，三产超过一产8.3个百分点。通过五年来的持续发展，主要经济指标在全省、全市位次不断前移，摆脱了多年落后垫底的局面。“小麦、花生、生猪”三张全国百强县名片更加响亮，粮食产量实现“十三连增”，11次荣获“全国粮食生产先进县”荣誉称号，生猪存出栏连续19年位居全省第一，连续18年成为全国花生生产第一大县。2015年被科技部定为“国家级农业科技示范园区（核心区）”，2016年先后获得“全省一二三产业融合发展示范县”、全省首批命名的“主要农作物生产全程机械化示范县”、“全省农机安全生产先进县”等荣誉称号，县产业集聚区被省政府评为全省“十快产业集聚区”。回顾这五年来，我们主要抓了以下几个方面工作：

围绕保增长促发展，项目建设取得突破。按照“上争、下促、外引、内培”的工作思路，依托农业资源优势，围绕食品和装备制造两大主导产业，招大引强。五年来，引进了君乐宝乳业、牧原集团、台湾巴布豆等一批知名企业，内培了华星铸造、机械制造产业园、古汉井醋等重点项目，其中，引进的君乐宝项目，半年内实现开工到投产，创造了君乐宝集团建厂新速度。初步形成了维维粮油产业园、机械制造产业园、温州特色食品产业园、正花有机食品产业园等园区。五年来，先后开工亿元以上项目111个，总投资498.2亿元。外贸出口连续增长，2016年出口额达到了1578万美元，年均增幅达到204.8%，居全市前列。同时，加大政策性项目资金争取力度，共争取中央、省各类政策性项目414个，总投资53.9亿元，其中争取政策性资金36.9亿元。

围绕抓建设补短板，城乡面貌明显改观。全面编制完善了县城总规，控制性详规和综合交通、市政设施、专业市场等13项专项规划，形成了系统完善的城乡规划体系。县城区规划面积扩大到50平方公里，建成区面积达到25平方公里，常住人口20万人，城镇化率达到35%。五年来，共投入城市建设资金86亿元，先后启动13个棚户区改造、“两河十路三入口”等城建工程，完成了护城河、慎水河治理美化工程和韦甲沟综合治理暨清水湾公园建设；建成了正阳公园、正阳广场，改造了文化广场；建设了新地春天、巨源城市花园等一批房地产项目，城市居民居住环境进一步改善。升级改造了中心大街等22条城区主干道路，打通城区道路6条，开通了4条城市公交，初步实现了路网循环。完成了县污水处理厂提标改扩建和第二污水处理厂新建工程，城市供排水、垃圾处理、照明、绿化等基础设施日臻完善。深入开展城市创建活动，实施了96条背街小巷和家属楼院路的硬化亮化美化工程，治理改造了万宝聚市场、钟鼓楼农贸市场、中心菜市场，改造公厕92座、旱厕759座，建设垃圾收集站22座、垃圾中转站14座。持续推进“双违”集中整治，共拆除各类违法建筑1362处，拆除面积18.2万平方米，违法违规建房势头得到遏制。通过加大投入和规范管理，城市形象不断提升，2012年至2016年，从创建市级卫生城、文明城到成功创建国家卫生县城、获得省级文明县城提名，实现了双跨越。五年来，持续改善农村人居环境，建立了投入、保洁、宣传、考评四项工作机制，截止2016年底，成功创建了149个达标村，65个示范村。2015、2016年连续两年被评为省改善农村人居环境先进县。

围绕上规模创特色，农业优势更加显现。坚持以农业供给侧结构性改革为导向，加快农业结构调整步伐，粮食产量实现“十三连增”。生猪存出栏突破400万头。花生种植面积保持150万亩，是全国小果型花生生产规模最大的产区。全县农业机械生产企业发展到38家，拥有各类花生机械达到16.54万台，实现了花生从种植、收获到加工的全程机械化。我县农业供给侧改革工作得到了省市领导的充分肯定。2016年，我县被选为全省农业结构调整暨三秋生产现场会观摩点，并在全市经验交流会上做了典型发言，央视七套以《黏土地上的奇迹》对正阳花生进行了专题报道。

围绕抓主导求实效，产业园区提质增效。五年来，县产业集聚区实现了规模由小变大、发展速度由慢变快、发展质量由低变优的转变。产业集聚区规划面积达到19.44平方公里，已建成10.2平方公里。建成了“六横八纵”的交通路网，电力、供排水、燃气、通信、照明、保障性住房等设施配套，承载能力进一步增强，形成了以食品和装备制造为主导的产业发展格局。止2016年底，入驻企业159家，其中规模以上企业119家，从业人员突破3万人，规模以上企业主营业务收入突破百亿。正阳君乐宝乳业被确定为省属重点企业，实现了我县没有省重点项目的零突破。

围绕破瓶颈强支撑，基础设施明显改善。综合交通体系建设成效显著。建成了京港澳高速正阳连接线，县城20分钟可上高速。大广高速连接线基本完工。先后投资3.3亿元，改造提升了县城至大林、县城至中心庙等5条干线公路，全县干线公路总里程超过185公里。息邢高速正阳段征地拆迁工作基本完成，已全面进场施工。全面实施农村公路三年行动计划，总投资2.7亿元，改建农村公路及危桥项目139个，修建乡道139.2公里、村道267.6公里。持续开展超限超载专项治理，通过建立检测站点、增加治超车辆和设备、全面禁止河道采砂等措施，实现路面治超和源头治超相结合。电力建设步伐加快。新建了1座220千伏变电站、2座110千伏变电站、8座35千伏变电站，扩建了110千伏变电站1座，对骨干电网、农村电网进行了改造升级。信息网络系统不断完善。全县移动网络覆盖率达到99.98%，城区主街道区域和乡镇集镇4G网络实现全覆盖。水利支撑能力明显增强。先后实施了小农水重点县、千亿斤粮食项目、农业综合开发等农田水利设施建设项目，解决了18.97万人的农村安全饮水问题。全面实施了蓝天、碧水、乡村清洁和河流清洁行动计划等环保治理工程，深入开展大气污染防治攻坚战。2016年，我县优良天数达到231天，位居全市第2位。

围绕保民生促和谐，社会事业全面发展。县财政用于民生支出逐步增加，2016年，全县用于教育、医疗等民生重点支出33.1亿元，占公共财政预算支出的81.7%。落实精准扶贫、精准脱贫基本方略，出台了扶贫对象精准识别、财政涉农资金统筹整合、脱贫成效考核等实施办法，抓住转移就业、产业扶持、社会保障等重点工作，强化教育、交通、医疗、水利、文化等基础建设，实现稳定脱贫。五年共实现45个贫困村、6.3万人稳定脱贫。五年新增城镇就业4.18万人，累计转移农村劳动力5.95万人次，城镇登记失业率1.34%。企业退休人员生活待遇和机关事业单位工作人员待遇不断提高。城乡居民养老保险制度从无到有，参保人数达到44.56万人。城镇居民医疗保险财政补贴标准由200元提高到420元，参保率达到99%。各类教育均衡发展，高考升学率进入全市先进行列，连续4年荣获全市“高中教学先进县区”，连续3年北大清华榜上有名。职业教育稳居全省先进位次，并跻身全国百强。落实残疾人“两项补贴”政策，社会养老、农村五保供养等基本社会服务体系日臻完善。五年来累计建成保障房8300套，实施危房改造8103户，建成村级卫生室143个，乡镇卫生院门诊病房楼15个。县医院门诊楼、县卫生监督所暨120指挥中心建成投入使用。社会治安形势总体良好，没有发生大的安全生产事故。信访形势总体平稳，社会大局保持和谐稳定。

围绕促改革求动力，政府职能不断转变。五年来，先后完成了食品药品、科技工信、卫生计生、文化广电、工商质监等行政管理体制改革，完成了交通运输执法体制改革，成立了交通运输综合执法局。整合组建了城市管理综合执法局。真阳办事处正式挂牌，实现了正阳县没有办事处的突破。积极开展招行引资，成立了正阳县金融工作办公室，农商银行成功改制，玉川村镇银行挂牌营业，金融保险事业稳步发展，金融风险整体可控。土地确权工作基本完成，农村土地承包经营权确权颁证工作全面展开，县乡村土地流转服务平台基本建成。新型农村经营主体发展迅速，全县农民专业合作社、家庭农场总数分别达到1247家和2640家，省级以上农业产业化龙头企业4家，国家级专业合作社5家。建立了政府“权责”清单制度。持续推进“简政放权、放管结合、优化服务”改革，不断优化公共服务，方便基层群众办事创业。

围绕抓两转促两提，自身建设不断加强。五年来，县政府始终坚持服从党的领导，转变政府职能、转变工作作风，进一步提高了行政效能和公务员素质，完善了政府重大事项集体决策、县政府领导班子周例会、重大事项报告等工作制度；修订了《正阳县人民政府工作规则》等一系列规章制度，建立了规委会、土委会、环保委员会等。坚持重大问题决策、重要人事任免、重大项目投资和大额资金使用集体决策并及时向县委常委会报告。扎实开展群众路线教育实践活动、“三严三实”、“两学一做”专题教育。认真纠正“四风”，落实中央八项规定，政府系统党风廉政建设不断加强。认真执行县人大常委会决议、决定，自觉接受人大及其常委会的法律监督、工作监督和政协的民主监督。五年来，共办理人大代表建议467件，政协委员提案1090件，办复率100%，人大代表、政协委员满意率达到97%以上。

五年来，统计、审计、粮食、气象、档案、供销、食品药品监督、计划生育、体育、史志、人防、民兵预备役、外事侨务、广播电视、民族宗教、消防、农场、保险、工会、红十字、妇女儿童等各项工作都取得了长足发展。

各位代表！五年的奋斗历程，是面对各种困难挑战的环境下，自我加压、负重奋进的历程；是实现加快发展、弯道超车的历程。成绩可喜可贺，经验更值得总结。认真归纳起来，主要是得益于以下几个方面：一是得益于始终坚持“以党建为引领，以党员为先锋”的工作思路。始终把加强和服从党的领导放在首位，严格按照县委提出的“五个二”工作思路，布局全县经济社会发展大局，充分发挥党组织的堡垒作用，在全县广大党员中开展“戴党徽、亮身份”活动，弘扬党员的先锋模范精神，全县上下营造出了敢于担当、敢于干事的良好局面，有力地推动了全县经济社会的发展。二是得益于始终坚持“聚精会神抓建设、一心一意谋发展”的指导思想。始终把加快发展作为解决正阳所有问题的关键点，把提高人民群众的生活水平作为满足人民群众需求的出发点，保持定力，咬定发展不放松，为全县经济社会持续发展提供了重要支撑。三是得益于始终坚持“集中力量办大事，统筹兼顾促发展”的工作方法。尽管财力有限，但我们始终坚持将重大基础设施建设作为营造发展新优势、加快新型城镇化发展的有效抓手，积极争取，倾注全力，加大投入，推进城市创建、县乡公路建设、高速公路建设、新老城区发展、农村基础设施等一批事关我县长远发展的重大项目，为聚积后发优势、实现经济社会持续健康发展打下了坚实基础。四是得益于始终坚持“开放包容纳百川，内外联动强合力”的聚力之策。始终将改革开放、招商引资作为“一把手工程”，围绕主导产业，坚持招大引强，全面优化投资环境，吸引知名企业立足正阳、做大做强，持续增强发展动力和后劲。五是得益于始终坚持“一张蓝图绘到底，一以贯之抓落实”的发展定力。五年来，按照“十二五”，尤其是“十三五”规划要求，细化目标任务，严格督查落实，咬定发展目标，不折不挠，持之以恒，不放弃，不动摇。六是得益于始终坚持“保障和改善民生，让发展成果惠及广大群众”的目标追求。以增强群众获得感为工作导向，千方百计加大民生投入，围绕脱贫攻坚及人民群众关心的切身利益问题，努力解决好教育、医疗、道路、城乡建设、环境卫生等工作，让群众分享改革开放的成果。

各位代表！五年来，我们迎难而上、负重前行，把发展作为解决一切问题的关键，一步一个脚印，一年一个台阶，取得了令人鼓舞的发展成绩。这是市委、市政府和县委正确领导的结果，是县人大、县政协监督、支持的结果，是全县人民迎难而上、拼搏进取、共同奋斗的结果！在此，我代表县人民政府，向在各条战线、各个岗位上辛勤工作、无私奉献的全县人民，向给予政府工作大力支持的人大代表、政协委员，向历任老领导、老同志，向人民团体、工商联和社会各界人士，以及关心、支持正阳发展的同志们、朋友们，表示衷心的感谢并致以崇高的敬意！

在肯定成绩的同时，也要清醒地看到，当前我县经济社会发展中还存在一些突出矛盾和问题。主要表现在：一是经济总量偏小。我县近几年经济虽然保持了快速增长，但基础差、底子薄、总量小的现状尚未得到根本扭转，一产比重大、二产比重小、三产发展不优的问题依然没有得到根本解决。特别是工业基础薄弱，企业总量小、个头小、链条短、竞争力弱，大项目、好项目少，稳增长、保态势的压力依然很大。二是农业基础设施薄弱。投入不足，历史欠账多，农业抗御自然灾害的能力不强，靠天吃饭的状况尚未得到根本改变。农业产业化程度不高，农业科技推广应用不足，农产品质量和效益不高，市场竞争力不强，农民来自于农业生产的收入增长缓慢。三是城镇化水平偏低。城镇化率低，建设欠账多，城市交通、供排水等基础设施功能不完善，环境脏乱、秩序混乱的问题还比较突出。四是群众关注热点问题解决不够。交通车辆拥堵问题还没有得到有效解决，比如正大路拉砂车辆过多，存在一些交通安全隐患，城市交通堵塞等现象依然没有得到根治，交通秩序有待进一步改善。农村养殖污染问题比较突出，农村生活垃圾处理、环境污染治理任务艰巨。另外，征地拆迁、城乡低保、特殊利益群体、非法集资等敏感问题，群众反映和诉求较多，信访形势依然严峻。五是作风建设需进一步加强。一些单位、部门工作人员懒政怠政，不负责、不担当、不作为、推诿扯皮、服务效率不高的问题还时有发生，项目推进慢、群众办事难现象依然存在。这些问题的存在虽然有一定的客观因素，但更多是我们主观努力不够的结果。对此，我们必须高度重视，强化措施，认真解决。

二、今后五年发展目标

各位代表！未来五年，是正阳大有可为的黄金发展期，也是全面建成小康社会、实现赶超发展、弯道超车的关键机遇期。我们一定要坚定信心，乘势而为，加快发展。一是中央经济工作会明确提出要适应、把握、引领经济新常态。从今年开始，国家将围绕供给侧结构性改革，“三去一降一补”等方面密集出台一系列促发展的政策措施，必然为我们争取更多项目资金支持带来更多机遇。二是长三角、珠三角“腾笼换鸟”速度加快，为我们开展招商引资、承接产业转移提供了难得机遇。新时期促进中部地区崛起计划、覆盖我县的大别山振兴发展规划、淮河生态经济带建设已纳入国家重大发展战略，加之我县息邢高速、安罗高速的快速建设及县域内交通路网建设的全面提升，我们的区位、交通、环境条件将得到极大改善，势必为我们带来难得的发展机遇。三是省委经济工作会上提出的发挥优势打好“四张牌”、“百城建设提质工程”、推进农业供给侧结构性改革等，都为我县发挥比较优势、加快发展转型提供了条件。四是经过近几年的不断投入和发展，正阳积累的优势和潜力将会得到全面释放。特别是近年来全县上下营造了风清气正、干事创业的良好政治生态，部分经济指标增幅已跨入全市，乃至全省先进行列，全县上下呈现出了发展氛围浓、发展环境优、发展势头好的可喜局面。所以，我们一定要保持好、巩固好这种来之不易的大好局面，坚定信心、乘势而为，努力开创正阳经济社会发展的新局面。

未来五年主要预期目标是：

——综合实力进一步提升。全县生产总值达到235亿元，年均增速8%；公共财政预算收入达到8.2亿元，年均增速9%；全社会固定资产投资达到283亿元，年均增速16%；社会消费品零售总额达到123亿元，年均增速13%；城镇化率达到45%，年均增长2个百分点，城镇人口达到38万人以上。

——结构调整进一步优化。深入推进供给侧结构性改革，促进产业升级，推进一二三产融合，持续提升产业竞争力。服务业增加值占生产总值比重提高到45%,三次产业结构形成三、二、一的格局；研究与开发经费占GDP比重提高到2%，高新技术产业增加值比重提高到28%，互联网普及率达到80%以上。积极调整农业结构，农业产业化能力进一步增强。以成功争取国家种养结合整县推进示范县为契机，在种植业、畜牧业结构调整过程中，探索现代农业发展道路。

——生态环境进一步改善。实施环境污染治理三大行动。持续开展大气污染防治攻坚战，到2020年，全县优良天数达到255天以上，重污染天气比例下降50%；万元GDP能耗、万元工业增加值用水量等完成市定目标。深入开展水污染防治攻坚战，水源地水质达标率达到98%以上，农村集中式饮用水源地达到85%以上，消除城市黑臭水体。全面治理农业面源污染和养殖污染，化肥农药实现零增长，规模以上养殖场实现达标排放。力争成功创建省级生态县。

——居民生活水平进一步提高。城镇居民人均可支配收入达到33580元，农村居民人均纯收入达到15730元，年均增长分别达到9%和10%；五年累计城镇新增就业4.5万人，转移农村劳动力5.5万人；农村安全饮用水普及率达到100%；严格贯彻落实《河南省改善农村人居环境五年行动计划（2016-2020年）》精神，所有行政村启动农村垃圾专项治理行动和污水处理，重点开展道路硬化、环境净化、乡村绿化、村庄亮化、农村文化五项工程，力争到2020年实现全县80%以上行政村创建省级达标村，40%以上行政村创建省级示范村，力争实现“双供”（农村实现供气，县城实现供热）。全县农村居民住房、饮水、用电和出行等基本生活条件得到明显改善，城乡居民生活宜居度不断提高。积极开展农村居民健康养老，实现老有所养。精准发力，确保到2018年底，减少贫困人口24795人（含两无人员），实现所有贫困村脱贫，贫困县摘帽。到2020年，全面实现人均纯收入达到全国人均纯收入平均水平，确保与全国同步进入小康社会。

——基础设施功能进一步完善。抢抓“一带一路”、大别山革命老区振兴发展和淮河生态经济带规划实施带来的机遇，以城市建设、综合交通网络、水利设施、能源和信息基础设施为重点，打造适度超前、功能完善、配套协调、高效安全的基础设施体系。绕城公路、息邢高速、安罗高速实现通车，正阳县城10分钟上高速，全面提升基础设施的现代化水平和支撑保障能力。

——文明法治水平进一步增强。社会主义核心价值观深入人心，深入开展群众性精神文明创建活动，做文明人、办文明事蔚然成风。法治政府基本建成，法律的实施、监督和保障体系进一步完善，社会更加和谐稳定。加快信用体系建设，进一步弘扬良好的思想道德风尚，全社会公平正义的氛围更加浓厚。

实现上述目标，必须坚持创新发展，培育发展新动力；必须坚持协调发展，构筑发展新格局；必须坚持绿色发展，建设美丽新家园；必须坚持开放发展，拓展发展新空间；必须坚持共享发展，创造幸福新生活。

各位代表！宏伟的目标召唤着我们，人民的期待激励着我们。我相信，经过五年的不懈努力，一定能够圆满完成全面建成小康社会这一伟大历史任务！

三、2017年主要工作二号首长

今年政府工作的总体要求是：全面贯彻党的十八大和十八届三中、四中、五中、六中全会精神，更加紧密地团结在以习近平总书记为核心的党中央周围，深入贯彻习近平总书记系列重要讲话精神和党中央治国理政新思想新路线，统筹推进“五位一体”总体布局和协调推进“四个全面”的战略布局，坚持稳中求进工作总基调，突出以推进供给侧结构性改革为主线，着力深化改革开放、推动创新，促进经济平稳健康发展和社会和谐稳定，以优异成绩迎接党的十九大胜利召开！

今年经济社会发展的主要预期目标是：生产总值增长8%以上，规模以上工业增加值增长10%，固定资产投资增长18%，社会消费品零售总额增长13%，地方公共财政预算收入增长9%，城乡居民收入增长10%，城镇化率提高2个百分点以上，新增城镇就业人口6000人以上，居民消费价格涨幅控制在3%以内，节能减排、生态环保完成市定目标。

各位代表！解决正阳所有问题的关键是加快发展。我们一定要紧紧围绕县委确定的工作思路和任务目标，凝心聚力，真抓实干，抢抓机遇，乘势而上，同心同德，共谋发展，推动全县经济社会发展再上新台阶，实现新突破！

今年着重做好以下八个方面的工作：

（一）以项目建设为抓手，加快赶超发展步伐。以项目建设作为推动经济发展的总抓手，强力推进项目建设。一要加强项目争取。正阳的发展靠项目，根本在项目，尤其是在经济新常态下更要积极向上争取项目。围绕国家重点支持的水利工程、粮食安全保障、城市建设、脱贫攻坚、生态环境等领域和我县经济社会发展的短板与薄弱环节，结合国家、省、市“十三五”规划和大别山革命老区振兴发展规划，争取更多项目，寻求资金支持。二要加大项目引进。突出产业招商。围绕农业优势资源，以农业招工业，补工业短板，依托主导产业延链补链，促进一二三产融合发展，使我县食品和装备制造业走上产业链条长、竞争优势大、发展质量优的现代化产业发展之路。突出招大引强。围绕主导产业，开展有针对性的定点招商、驻地招商、精准招商。2017年力争引进投资20亿元项目1个，10亿元项目1个，5亿元项目2个，3亿元项目4个，投资亿元以上项目20个。突出多领域招商。在积极引进工业项目的同时，围绕专业市场、商业贸易、现代物流、休闲娱乐等服务业和养老、城市基础设施等领域，积极引进一批好项目。三要加快项目建设。对2017年的重点建设项目，排出开工时间表，明确建设任务和时间节点，确保早开工、早建成、早投产、早收益；对进展缓慢的项目，认真分析原因，研究对策，加快推进。

（二）以打造宜居城市为核心，加快新型城镇化进程。牢牢把握“百城建设提质工程”机遇，努力打造宜居宜业、富有活力、特色鲜明的现代化城镇。一要严格执行规划。按照“规划先行、谋定后动”的工作思路，对县城各项规划进行完善，严格执行县城规划管理，发挥规划的调控、引领、约束作用，维护规划的严肃性和权威性。二要加快建设步伐。重点实施老城区功能提升和新城区设施完善，注重解决老城区北部交通拥堵问题，提高城市宜居度。加快实施江国大道向西等打通工程；完成正阳大道城区段等改扩建工程，升级改造慎水河路等5条新城区道路；进一步完善提升韦甲沟治理暨清水湾公园、正阳公园和古佛文化园建设，完成江国公园和东南出入口景观工程建设，启动慎水河中支生态治理和水源地森林公园规划建设，为城市居民提供优美生活环境；统筹谋划供排水、通讯、燃气等管网建设，完善城区供水体系，实现24小时供水。启动静脉产业园建设和第二污水处理厂中水回用工程。三要加强城市管理。以“四城联创”为抓手，推动城市精细化、规范化管理。继续推进民巷和家属楼院的硬化、美化、亮化工程，进一步完善县城垃圾收集点、公厕、绿地基础设施，在巩固创建成果的同时争创省级园林城市。实行县城环卫社会化服务。加快欧蓓莎国际商城建设，启动二级客运站、货车4S园区等专业市场建设。积极推进“数字化城市管理”建设，加大城市管理执法力度，突出城市环境、交通秩序、废旧物品收购等专项治理，解决私搭乱建、占道经营、乱涂乱画、乱堆乱放等突出问题，实现城市规范化管理。始终保持“双违”整治的高压态势，减少“双违”案件的发生。四要加快现代服务业发展。重点推进现代物流、现代金融、信息服务、文化旅游、健康养老等五大主导产业发展，积极引进集商贸、物流、办公、超市、娱乐于一体的现代化服务商业区，实现集聚发展，带动我县服务业加快转型升级。2017年力争4家知名电商企业入驻县产业集聚区。启动花生食品城和深豫物流园建设。推进中原国际城、伟品市场建设。五要促进房地产市场平稳健康发展。加快正阳信赖城市综合体、巨源花园城市综合体等房地产项目建设进程；加快推进吴湾、田楼等城中村和皮件厂等棚户区改造项目。

（三）以农业结构调整为重点，加快推进三农工作。以加快推进农业供给侧结构性改革为主线，围绕“四优四化”，进一步优化农业结构，加快一二三产融合发展。一要努力调整农业结构。大力发展优质小麦、优质花生、优质草畜和优质瓜果。与花生科研院所开展深入合作，加快花生良种繁育基地建设步伐，发展具有正阳特色的彩色花生和高油酸花生。立足于君乐宝现代化旅游观光示范牧场落地正阳的优势，鼓励农民进行粮改饲，发展食草性动物养殖业。加快推进牧原养殖基地建设，鼓励支持养羊、养禽等规模化养殖，优化畜牧业结构。二要积极发展新型农村经营主体。重点扶持发展一批省级农业产业化龙头企业、农产品加工企业和新型农村经营主体。全年新建农民专业合作社100家以上，家庭农场50家以上。加快农村土地承包经营权确权发证步伐，在土地流转、政策扶持等方面给予新型经营主体全力支持，做大做强，培育集群。三要加快一二三产融合。依托我县作为国家级农业科技示范园区核心区的优势，大力发展智慧农业，培育壮大农村新产业新业态。围绕花生产业，以正阳君乐宝乳业、机械制造产业园、正花有机食品产业园为依托，拉长产业链条；以正阳花生信息产业园为基础，打造全国花生信息交易平台，加快一二三产业融合发展步伐。四要积极抓好“创森”工作。按照市委、市政府关于创建国家森林城市的统一部署，突出廊道绿化、精品林网、生态防护林等重点，完成造林任务10万亩，实现林木覆盖率达到26%的目标任务，为2018年全市通过国家森林城市验收打下基础。五要切实改善农村人居环境。今年要实现创建170个人居环境达标村、85个示范村的目标。围绕改善农村人居环境，继续加强农村道路、垃圾治理等工程建设。同时，加大美丽乡村建设力度，打造具有地方特色的乡村小集镇。

（四）以产业集聚区建设为载体，增强工业发展后劲。以新修编规划为引领，拓展空间，完善功能，优化布局，着力提升产业集聚区的规模和层次。一要加大投入。进一步建设和完善集聚区内水、电、路、气、通讯、供热等基础设施，提高承载能力。加快集聚区内循环路网体系建设。二要做大园区。加快雏鹰枫桦生态食品产业园、圣诞礼品产业园、机械制造产业园、正花有机食品产业园等重点园区建设进度，尽快形成竞争优势明显、支撑带动作用强劲的主导产业和产业集群，打造一二三产融合的产业集聚区。三要集约节约用地。结合城中村改造，加大产业集聚区内村庄搬迁安置力度，为集聚区的发展储备土地，同时，积极盘活存量建设用地，提高土地利用率，实现土地集约节约利用。四要壮大外向型经济。认真落实出口奖励政策，重点培育以华星机械、三合球铁、精工铸造等为主体的传统出口基地。发展壮大圣诞礼品产业，培育新兴外贸出口企业。

（五）以脱贫致富为目标，强力推进脱贫攻坚。继续在“精准”上下功夫，切实把“精准”贯穿于脱贫攻坚全过程，做到精准识别、精准施策、精准退出、精准考核。2017年实现24个贫困村、4424户、10304人稳定脱贫。一要突出产业扶贫。以“产业扶贫年”活动为载体，学习借鉴台前县、范县、滑县等地先进经验和做法，按照“一村一品”或“数村一品”的要求，重点围绕能够发挥当地资源优势、切合产业发展、市场前景好、适于贫困户发展的高效种植业、规模养殖业、农产品加工储运等项目，建设“扶贫基地”，扶持各具特色的扶贫产业。积极探索“资源变资产、资金变股金、农民变股东”模式，增加经营性收入和财产性收入，提高贫困户稳定收入水平。同时，充分发挥金融、保险对脱贫攻坚的助推作用，实行小额信贷贴息资金补贴，为产业扶贫保驾护航。积极做大担保平台，撬动更多社会资本用于扶持新型农村经营主体，带动贫困户脱贫。二要统筹整合资金。统筹整合使用涉农项目资金，重点实施贫困村村室、通村公路、文化服务中心、卫生室、安全饮水、教育等基础设施建设，助推脱贫攻坚。三要鼓励自主脱贫。根据贫困人口、贫困家庭的情况，有针对性的进行技术培训，宜农则农，宜工则工。通过金秋助学、大病保险等形式，加大教育、健康等扶贫力度，为实现自主脱贫增添内生动力。认真做好社会保障兜底脱贫工作，对贫困人口实现全覆盖。四要加强社会帮扶。开展广泛的社会动员，号召社会各阶层人士、社会团体共同参与脱贫攻坚，让更多的社会力量投入到扶贫工作当中。

（六）以推进改革创新为引领，增强内生发展动力。一是继续推进行政管理体制改革。加快转变政府职能，继续清理取消县级行政审批事项。全面推行权力清单制度。积极推进“互联网+政务服务”，统筹推进机关事业单位养老保险制度改革和工资收入分配制度改革。二是继续推进财税金融改革。改进预算管理制度，严格规范预算执行，完善事权与支出责任相适应的财政管理制度。优化财政收入结构，积极培植新税源，提高税收在财政收入中的比重。深化投融资体制改革，加快政府投融资平台市场化转型，提升融资能力。积极开展政银企对接，解决小微企业抵押难、担保难、融资难问题。严厉打击非法集资行为，防范和化解金融风险。积极稳妥推进国有企业改革。三是继续深化医药卫生体制改革。统筹推进医疗、医保、医药联动改革，推进公立医院综合改革。进一步完善基本药物制度，全面取消药品加成，破除以药养医。加快综合医改、分级诊疗试点，积极推进家庭医生签约服务；健全重特大疾病保障制度，促进基本公共卫生服务均等化。四是继续深化农村体制改革。把深化农村改革和创新农业经营体系、产业体系、服务体系、政策体系结合起来，统筹推进，加大土地流转力度，激发农村经济活力、调动农民积极性。6月底前全面完成乡镇土地承包经营权确权颁证工作。五是继续深化科技体制改革。选择引进一批市场前景好、研发能力强的高新技术产业落户正阳，支持其发挥自身优势引领全县企业创新发展。依托产业集聚区、骨干企业、高校和科研院所，逐步建立高层次人才创新创业基地，集聚高层次创新创业人才和团队。

（七）以强化基础设施为支撑，培育发展竞争新优势。按照省委提出的打好“四张牌”的政策要求，不断强化基础能力建设。一是道路建设。优化施工环境，为加快息邢高速正阳段建设创造条件，确保如期通车。启动息邢高速连接线建设，配合做好安罗高速的前期工作；加快干线公路的大修进度，完成正陡路大修改造；加快农村公路改建和通村公路建设，全年新修县乡公路29.6公里，通村公路83公里，桥涵8座；启动县城新区二级客运场站建设；增加公交车辆投放，进一步改善城市居民出行条件；继续保持高压态势，加大旱砂场治理力度，狠抓超限超载治理。加强交通安全宣传教育，完善公路基础设施，规范交通秩序，减少交通安全事故。二是农田水利建设。新建高标准农田3.4万亩，解决涉及16个贫困村、52192人口的安全饮水问题。实施吕河邱店至王勿桥段、清水河后王庄至夏王庄治理工程，不断提高水利支撑作用。三是电力设施建设。新建110KV铜钟输变电工程，寒冻、陡沟、袁寨35KV输变电工程和110KV铜钟变电站35KV配出工程，强化电力服务经济社会发展的保障作用。四是通讯设施建设。充分利用移动、联通、电信等通讯平台，加快我县信息化进程，逐步实现县乡通讯网络全覆盖，提升“互联网+”等各项通讯设施对经济发展的基础性支撑能力。

（八）以保障和改善民生为根本，实现社会和谐稳定。坚持民生优先，尽力而为的指导思想，着力保基本、兜底线、促公平，努力提高就业、养老、教育、医疗等公共服务水平和质量，不断改善人民生活。一要加大投入。在积极向上争取民生项目的基础上，县本级财政预算支出继续向民生倾斜，实现投入稳定增长。二要保障改善民生。深入实施全民技能振兴工程和农民职业技能提升计划，统筹抓好农村转移劳动力、城镇困难人员就业、就业扶贫工程，鼓励支持农民工、大学生返乡创业。全年新增城镇就业6000人以上，农村劳动力转移就业8500人，失业人员再就业1900人。做好全县社会保险的参保入户登记工作，实现社会养老保险参保全覆盖。加快县就业和社会保障服务中心建设。完成县儿童福利院项目建设，实施养老托老中心和老年活动中心建设项目，新建2个乡镇敬老院。对城乡低保重新申报、精准认定，特困人员供养复核认定，切实做好城乡医疗救助、临时救助等工作。落实好残疾人“两项补贴”。全年改造农村危房1300户，提高危房改造质量。三要促进教育文化卫生发展。继续做好教育教学工作，推动义务教育均衡发展、高中教育优质特色发展、学前教育优质普惠发展。启动91所公办学校的全面改薄项目，加快实验中学、第八小学、第十四小学的迁建、新建工作；改造正阳一高、二高学生餐厅；推进正阳县职业中专扩建、正阳高中二期续建、正阳县全民健身中心工程建设，积极争取职业教育实验实训基地项目。加快现代双语学校、太极学校项目建设。每个脱贫村要有1所幼儿园，小学全部配套改造。积极创建国家级卫生乡镇。完成中医院病房楼建设，争取中医院门诊楼项目开工。加快妇幼保健院门诊综合楼、疾控中心综合楼项目建设。新建40个标准化村卫生室。深入开展爱国卫生工作，落实计划生育全面二孩政策，改善妇幼健康服务，探索医养结合模式，应对人口老龄化问题。加快实施县文化体育活动中心和8个乡镇、41个村级综合文化服务中心建设。继续开展“文化惠民、送戏下乡”活动，戏曲演出不少于300场，基本覆盖全部行政村。四要严格土地执法。进一步加强土地卫片执法监管，严格执行土地管理政策，落实县直单位、乡镇党委政府耕地保护责任制，继续保持打击违法违规用地高压态势，强化执纪问责，彻底打破土地卫片执法建了拆、拆了建的“怪圈”。五要加大环保整治力度。聚焦重点区域、重点行业，开展大气污染防治攻坚战。启动环境治理规划建设，全面完成各项考核目标任务，确保PM10年均浓度在100微克/立方米以下、PM2.5年均浓度在60微克/立方米以下、优良天数为210天以上。下决心打好水污染防治攻坚战，以实现水质达标、治理黑臭水体、保障饮水安全为重点，系统推进水污染防治和水生态保护。紧紧抓住全国种养结合整县推进试点县的机遇，把养殖业污染治理与水污染防治有效结合起来，实现生态、经济效益双赢。六要维护社会大局稳定。深入贯彻新《安全生产法》，加强安全生产、质量技术监督和食品药品安全监管，规范食品药品生产经营行为。加大火灾隐患排查力度，坚决遏制重特大事故发生。加强社会治安综合治理，预防大的治安事件和刑事案件的发生。切实做好信访稳定工作，高度关注征地拆迁、农民工工资、特殊群体等热点问题，依法依规妥善处置，确保社会和谐稳定。

加强国防教育和国防后备力量建设，支持驻正武警部队、民兵和人民防空建设，深入开展双拥共建，推进军民融合发展。支持工会、共青团、妇联、残联等人民团体发挥桥梁纽带作用，注重发挥工商联等社团作用。认真做好统计、审计、粮食、民族宗教、广播电视、外事侨务、农场、供销、档案、史志、气象等工作，促进社会全面进步。

四、加强政府自身建设

新时期新常态对政府加快职能转变、顺应时代潮流提出更高要求，全县政府系统要进一步解放思想，与时俱进，开拓创新，依法行政，从严治政，努力建设学习、法治、服务、创新、廉洁型政府。

（一）建设学习型政府。一是加强学习。不断加强党的理论学习，认真学习和领会十八届三中、四中、五中、六中全会精神和习近平总书记关于治国理政的新思想新要求，用科学的理论和先进的思想补足精神之“钙”、筑牢思想之“魂”。二是增强党性。始终在思想上、政治上、行动上同以习近平同志为核心的党中央保持高度一致，切实增强“四个意识”特别是核心意识、看齐意识，思想上深刻认同核心、政治上坚决维护核心、组织上自觉服从核心、行动上坚定紧跟核心。重点加强基层党组织建设，全面提高基层党组织的凝聚力和战斗力。三是提升能力。围绕事关我县经济社会发展的重大问题开展针对性的专题学习，把学习的成效转化为能力的提升，增强驾驭全局、应对复杂局面的能力，特别是要提高解决制约正阳经济社会发展“瓶颈”的能力。

（二）建设法治型政府。一是始终把依法行政、依法办事作为最高行为准则，贯穿于政府决策、执行的全过程，健全依法决策机制。二是推进服务型行政执法体系建设，全面落实行政执法责任制。组织开展法治培训，强化尊法学法守法用法意识，提高执法人员法治素质和法治能力。三是依法接受人大及其常委会法律和工作监督，主动接受政协民主监督。严格执行人大及其常委会决定，认真办理人大代表建议和政协委员提案，拓宽参政议政渠道，广泛听取民主党派、工商联、无党派人士、人民团体意见。自觉接受社会监督、舆论监督和司法监督。

（三）建设服务型政府。一是深化简政放权。继续精简行政审批事项，建立政务服务平台，全面加强权责清单动态管理，推行在线审批、模拟审批，实行网上受理、办理、监管“一条龙”服务，推广“互联网+政务服务”，让企业和群众办事更便捷。二是强化服务职能。强化社会管理和公共服务职能，提高主动服务意识和服务水平，把更多精力放在服务企业、监管市场和解决民生上，让公共资源更多地向民生领域倾斜，努力为人民群众提供更好、更优、更均衡的公共产品服务。三是切实转变作风。全面加强政府作风建设，牢固树立为民服务意识，落实中央八项规定。继续开展“懒政怠政为官不为问责”活动，杜绝“门好进、脸好看、事不办”现象。重点加强项目建设、精准扶贫、双违双超、城乡低保等重点工作和民生领域的监督检查，聚焦监督执纪问责，不断提高行政效能，提升为民服务水平和效率。

（四）建设创新型政府。一是大胆创新。要坚持解放思想、与时俱进，改变影响发展的思维定势，革除束缚发展的体制弊端。要结合实际，在总结我县近年来的经验、教训、得失中，发现规律、找出“症结”，创造性地开展工作。二是提高标准。强化赶超发展的紧迫感和责任感，摒弃过去那种“过得去”、“随大流”的工作标准和工作要求，树立标杆意识和大局意识，争先进位，争创一流。以更高的工作标准、更严的工作要求、更快的工作效率提高政府的执行力。三是勇于担当。要善于发扬敢闯敢试、敢为人先的精神，摒弃“宁愿不做事，也要不出事”的思想，凝心聚力，真抓实干。要强化督查问责，严厉整肃庸政懒政怠政行为。

（五）建设廉洁型政府。一是加强教育。采取上廉政党课、警示教育等形式，加强政府系统广大干部职工，特别是执法人员经常性廉政教育，做到警钟长鸣，不越“雷池”，不踏“红线”，在思想上筑牢防腐拒变的“堤坝”，逐步打造一支廉洁奉公的公务员队伍。二是强化监督。积极推进政务公开，把公开透明作为政府工作的基本制度，大力推进财政预算决算、公共资源交易等重点领域信息公开，实现政府决策的透明化、科学化，让群众看得到、听得懂、能监督。三是严守规矩。严守政治纪律和政治规矩，自觉服从县委领导，自觉接受人大、政协监督。认真落实党风廉政建设主体责任和“一岗双责”，全面落实中央八项规定，一以贯之纠正“四风”。继续保持惩治腐败高压态势，聚焦重点领域，严肃查处侵害群众利益的不正之风和腐败问题。

各位代表！

梦想承载希望，实干开创未来。过去的五年，我们战胜了各种困难和挑战，取得了辉煌的业绩。未来的五年，形势催人奋进，事业任重道远。我们要牢牢把握当前良好的发展形势，在县委的坚强领导下，在县人大、县政协，特别是各位人大代表、政协委员的监督支持下，紧紧抓住这一轮经济发展机遇，紧跟时代发展步伐，解放思想、开拓进取、奋力拼搏、务实重干，以时不我待的紧迫感和赶超发展的责任感，努力创造经得起实践、人民和历史检验的新业绩，不辜负全县人民的重托，为实现富民强县、赶超发展而努力奋斗！
